# Real-world flash glucose monitoring in Brazil: can sensors make a difference in diabetes management in developing countries?

**DOI:** 10.1186/s13098-019-0513-z

**Published:** 2020-01-07

**Authors:** Luis Eduardo P. Calliari, Marcio Krakauer, Andre Gustavo Daher Vianna, Yashesvini Ram, Douglas Eugenio Barbieri, Yongjin Xu, Timothy C. Dunn

**Affiliations:** 1 Santa Casa de Sao Paulo School of Medical Sciences, São Paulo, Brazil; 2 Centro de Pesquisa Clínica do Grupo Leforte, São Paulo, Brazil; 3 Centro de Diabetes Curitiba, Curitiba, Brazil; 4 Abbott Diabetes Care, Alameda, CA USA

**Keywords:** Flash glucose monitoring, Blood glucose monitoring frequency, Real-world data, Glycemic measures, Continuous glucose monitoring

## Abstract

**Background:**

New technologies are changing diabetes treatment and contributing better outcomes in developed countries. To our knowledge, no previous studies have investigated the comparative effect of sensor-based monitoring on glycemic markers in developing countries like Brazil. The present study aims to evaluate the use of intermittent Continuous Glucose Measurements (iCGM) in a developing country, Brazil, regarding (i) frequency of glucose scans, (ii) its association with glycemic markers and (iii) comparison with these findings to those observed in global population data.

**Methods:**

Glucose results were de-identified and uploaded to a dedicated database when Freestyle Libre™ readers were connected to an internet-ready computer. Data between September 2014 and Dec 2018, comprising 688,640 readers and 7,329,052 sensors worldwide, were analysed (including 17,691 readers and 147,166 sensors from Brazil). Scan rate per reader was determined and each reader was sorted into 20 equally-sized rank ordered groups, categorised by scan frequency. Glucose parameters were calculated for each group, including estimated A1c, time above, below and within range identified as 70–180 mg/dL.

**Results:**

In Brazil, reader users performed an average of 14 scans per day, while around the world, reader users performed an average of 12 scans per day (p < 0.01). In Brazil dataset, those in the lowest and in the highest groups scanned on average 3.6 and 43.1 times per day had an estimated A1c of 7.56% (59 mmol/mol) and 6.71% (50 mmol/mol), respectively (p < 0.01). Worldwide, the lowest group and the highest groups scanned 3.4 times/day and 37.8 times/day and had an eA1c of 8.14% (65 mmol/mol) and 6.70% (50 mmol/mol), respectively (p < 0.01). For the scan groups in both populations, the time spent above 180 mg/dL decreased as the scan frequency increased. In both Brazil and around the world, as scan frequency increased, time in range (TIR) increased. In Brazil, TIR increased from 14.15 to 16.62 h/day (p < 0.01). Worldwide, TIR increased from 12.06 to 16.97 h/day (p < 0.01).

**Conclusions:**

We conclude that Brazilian users have a high frequency of scans, more frequent than global data. Similarly to the world findings, increased scan frequency is associated with better glycemic control.

## Background

The number of people globally with diabetes is increasing, particularly in developing countries [1]. Effective glycemic control is essential for minimizing microvascular and macrovascular complications associated with diabetes. A1c is currently the gold standard for maintaining glycemic control as it has a strong predictive value for diabetes complications [2]. However, A1c does not provide a measure of glycemic variability or hypoglycemia [2]. Moreover, a range of mean glucose values and glucose profiles can be associated with a single A1c measure [3]. More recently, time in range (TIR) has been identified as a glycemic metric that captures both individual variability in glucose profiles and hypoglycemia [4] and is associated with the risk of microvascular and macrovascular complications [5, 6]. The advent of continuous glucose monitoring (CGM) has provided opportunities to analyze patient data in greater detail and aid in both standardizing and achieving glycemic targets [7]. Recently a consensus in TIR was published that emphasized the utility of TIR as a useful and appropriate clinical target [8].

Regularly monitoring blood glucose is essential for obtaining and maintaining glycemic targets. Previous studies have shown a strong correlation between the increased Self-monitored blood glucose (SMBG) frequency and increased glycemic control [9]. However, repeated collection of personal blood glucose measures can be difficult to maintain long-term, inconvenient and painful [10, 11]. The FreeStyle Libre™ flash glucose monitoring system enables patients to more conveniently assess their glucose readings, leading to a greater frequency of glucose testing. Recent research demonstrated that flash glucose monitoring in real world conditions allows for more frequent glucose checks associated with improved glycemic markers including increased time in range [12, 13].

While it was estimated that in 2017 there were 425 million people around the world living with diabetes, there are key differences between developed and developing countries [14]. Population-based surveys have shown that communities that have experienced westernization and urbanization associated with lifestyle change are at an even higher risk for diabetes, diabetes is twice as prevalent in urban settings compared to rural settings [14]. Brazil is a developing country that is rapidly urbanizing [15, 16]. Between 2017 and 2045 the Brazilian population with diabetes is expected to increase by 74% [14]. Currently, Brazil is the sixth most populous nation in the world with the fourth greatest prevalence of diabetes. In 2017, there were 12.5 million adults in Brazil living with diabetes [14]. Though developing nations bear a greater burden of diabetes compared with developed nations, to date, no studies have investigated the comparative effect of sensor-based monitoring on glycemic markers in developing countries like Brazil, as compared to the rest of the world in a real-world setting [17].

The aim of the present paper is to investigate the use of flash glucose monitoring in real life clinical practice both worldwide and in Brazil specifically over a period of 52 months to evaluate (i) frequency of glucose scans, (ii) its association with glycemic markers and (iii) comparison with these findings to those observed in global population data.

## Materials and methods

### Sensors and readers

The FreeStyle Libre™ flash glucose monitor is a needle-based sensor inserted below the skin that monitors glucose in interstitial fluid for up to 14 days. A dedicated reader is used to scan the sensor at any time to collect the current glucose, glucose trend, and up to 8 h of glucose readings automatically stored every 15 min. When connected to PC-based software with an active internet connection, the reader’s 90-day memory is de-identified and uploaded to a database. The report software, available for free download, includes an agreement that de-identified data will be collected at each internet-connected use of the software. From September 2014 to December 2018, this database collected a data set of glucose readings from 688,640 readers and 7,329,052 sensors, of which 17,691 readers and 147,166 sensors were collected from Brazil since it was launched, in 2016. Over 95% of the readers came from 26 countries on 6 continents with at least 2000 readers. The top five countries were Germany, France, Japan, United States, and Italy. Together these five countries contributed with approximately 59% of all readers. All data included in this study were deidentified and anonymous. No demographic or personal data regarding users was available to the authors of this paper.

### Scanning details

Scanning frequency for each sensor was calculated by counting the number of scans divided by duration of sensor use according to recorded start and end times. Scanning frequency per reader was assessed by calculating mean scans of all its sensors followed by determining cumulative frequency distribution and summary metrics (mean, median and IQR). To understand the daily patterns of scanning, frequency of scans by hour of the day was evaluated.

### Glycemic measures analyzed

The analysis required each sensor have at least 120 h of automatically-stored readings (480 readings) to ensure reliable glucose control measures. Data from all sensors belonging to the same reader were combined and calculated as the mean of all sensor measures. The readers were rank-ordered by scan frequency and allocated to 20 equally-sized groups. Each of the 20 groups consisted of 34,432 readers and 885 readers for the world and Brazil respectively. Glucose measures assessed included time in range (defined as glucose between 70 and 180 mg/dL), time in hyperglycemia (> 180 mg/dL) and time in level II hypoglycemia (≤ 54 mg/dL). We have chosen to focus on time below 54 mg/dL because the International Diabetes Study Group has designated this threshold as clinically significant biochemical hypoglycemia that has serious clinical and health-economic consequences and does not occur under physiological conditions in nondiabetic individuals. Unlike 70 mg/dL, 54 mg/dL is an unequivocally hypoglycemic value [18]. Finally, mean glucose was converted into estimated HbA_1c_ by the method accepted by international professional diabetes societies [19] and was also analyzed. The glucose control measures were inspected as a function of the ten scan-frequency groups of readers, and comparisons between scan frequency groups were evaluated.

### Statistical analysis

The cumulative frequency of scan rates was calculated for each 5% of available readers, and descriptive statistics were calculated. The frequency distribution of scans by hour of the day was inspected for scanning patterns across the day.

Given the large number of readers, 20 equally-sized groups (or bins), divided along scan rate, were analyzed by descriptive measures (mean and standard error) of glycaemic metrics. Statistical comparisons across the bins were performed by one-way analysis of variance (ANOVA), and the span of glycaemic measures and relative changes were reported from the lowest to highest scan rate bins.

The database was analyzed by structured query language routines, and further summarized by KNIME (http://www.knime.org) and R statistical package (http://www.r-project.org). In view of the large sample size and multiple comparisons, only p < 0.01 was considered statistically significant. Confidence intervals were calculated for each group least square mean of each measure for each scan rate group, and comparisons were made across the scan groupings.

## Results

### User base

The analysis set (Table 1) included 688,640 readers (17,691 readers from Brazil) with 7,329,052 sensors (147,166 sensors from Brazil) spanning 2.14 billion monitoring hours (43.2 million monitoring hours for Brazil), 8.55 billion automatically recorded glucose readings (173 million automatically recorded glucose readings from Brazil), and 1.1 billion sensor scans (26.5 million sensor scans from Brazil).

**Table 1:** Data collected from Sep 2014 through Dec 2018 (in absolute numbers)

	Brazil	All countries
Readers	17,691	688,640
Sensors	147,166	7,329,052
Glucose scans	26.5 million	1.10 billion
Monitoring hours	43.2 million	2.14 billion
Automatically-recorded glucose readings	173 million	8.55 billion

### Frequency

**Fig. 1:**
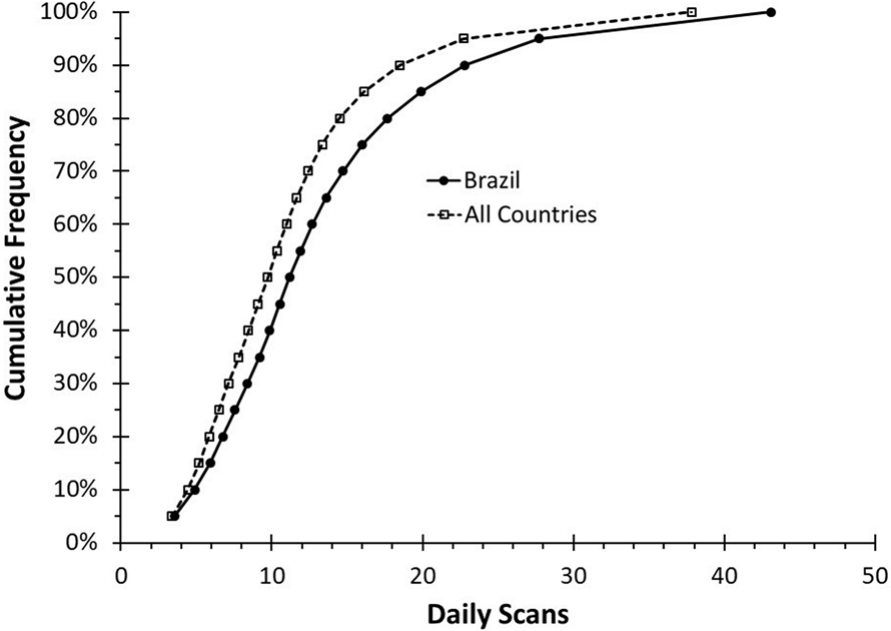
Daily scans per reader: a comparison of cumulative frequency by number of daily scans for users of the CGM device worldwide compared users in Brazil. The scan frequency of Brazilian users was significantly (p < 0.01) greater than the scan frequency of users worldwide

In Brazil, reader users performed an average of 14 scans per day, with a median (IQR) of 11 (8–16) daily glucose scans (Fig. 1). Around the world, reader users performed an average of 12 scans per day, with a median (IQR) of 10 (7–13) daily glucose scans (Fig. 1). For both the bins with the lowest and highest scan frequencies, users in Brazil scanned more frequently than users around the world (Table 2). Mean scan frequency for the lowest bin was 3.56 scans per day in Brazil and 3.40 scans per day around the world. Mean scan frequency for the highest bin was 43.07 scans per day in Brazil and 37.81 scans per day around the world. Overall, the scan frequency of Brazilian users was significantly (p < 0.01) greater than the scan frequency of users worldwide.

**Table 2:** Binned mean scan frequency (scans/day)

Bin N^o^.	Brazil	All countries
1	3.56	3.40
2	4.91	4.45
3	5.91	5.19
4	6.75	5.86
5	7.56	6.51
6	8.38	7.15
7	9.17	7.80
8	9.87	8.45
9	10.52	9.10
10	11.18	9.72
11	11.92	10.34
12	12.69	10.97
13	13.62	11.65
14	14.73	12.42
15	16.00	13.33
16	17.66	14.51
17	19.88	16.12
18	22.80	18.51
19	27.69	22.69
20	43.07	37.81

### Relationship between frequency of testing and glycemic parameters

The glycemic parameters analyzed in this study included the estimated A1c, time in hyperglycemia (hours per day above 180 mg/dL, time in range (TIR) (hours per day 70–180 mg/dL, and time in hypoglycemia (minutes per day at or below 54 mg/dL).

### Estimated A1c

**Fig. 2:**
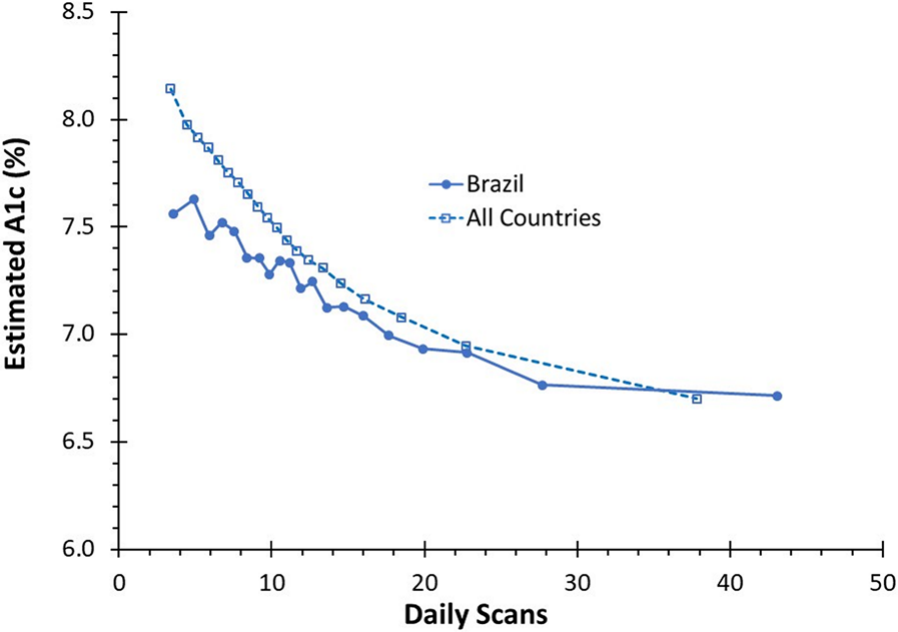
Estimated HbA1c: a comparison of estimated HbA1c by number of daily scans for CGM users worldwide compared to users in Brazil

In both populations, the mean estimated A1c reduced as the scan frequency increased (Fig. 2). In the Brazil dataset, those in the lowest group scanned on average 3.56 times per day had an estimated A1c of 7.56% (59 mmol/mol) (95% CI 7.44–7.68%, 60 mmol/mol, 33–97 mmol/mol) while those in the highest group scanned on average 43.1 times per day had a mean estimated A1c of 6.71% (50 mmol/mol) (95% CI 6.63–6.80%, 50 mmol/mol, 49–51 mmol/mol) (p < 0.01). In the worldwide dataset, those in the lowest group scanned an average 3.4 times per day and had an estimated A1c of 8.14% (65 mmol/mol) (95% CI 8.12–8.16%, 65 mmol/mol, 95% CI 65–66 mmol/mol) while those in the highest group scanned on average 37.8 times per day had a mean estimated A1c of 6.70% (50 mmol/mol) (95% CI 6.69–6.71%, 50 mmol/mol, 95% CI 50–50 mmol/mol) (p < 0.01). The estimated A1c of the bin with the lowest scan rate was clinically and significantly lower in Brazil compared to the rest of the world (7.16% lower, p < 0.01). However, there were no significant differences between the estimated A1c of the bin with the highest scan rate for Brazil compared to the rest of the world (p = 0.74).

### Time in hyperglycemia

**Fig. 3:**
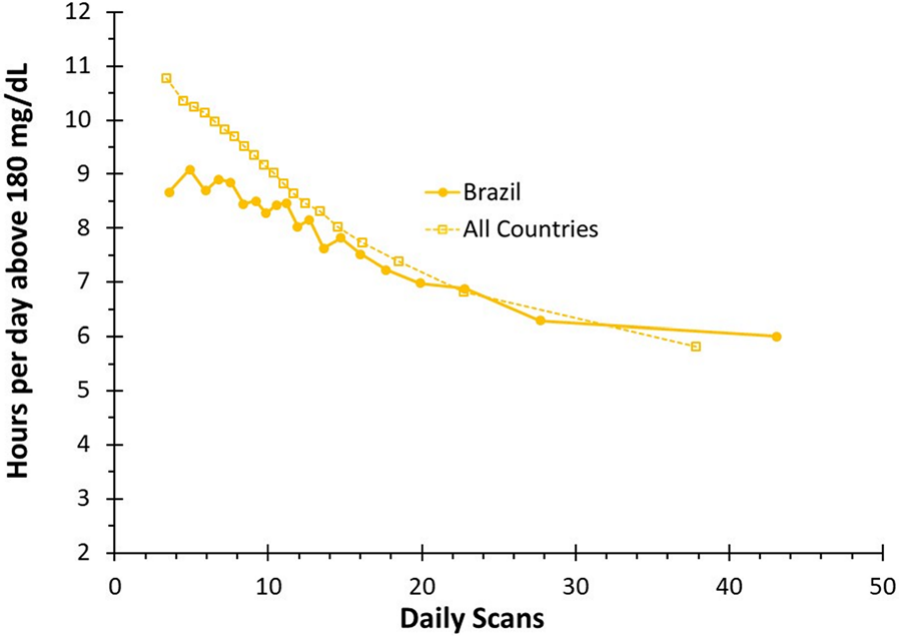
Time above 180 mg/dL: a comparison of mean hours per day in hyperglycemia by number of daily scans for CGM users worldwide compared to users in Brazil

For the scan groups in both the Brazilian and world populations, the time spent above 180 mg/dL decreased as the scan frequency increased (Fig. 3). In Brazil, the group with a mean scan rate of 3.56 times per day spent an average of 8.66 h/day in hyperglycemia (95% CI 8.27–9.06 h/day) while the group with a mean scan rate of 43.07 times per day spent an average of 6.00 h/day in hyperglycemia (95% CI 5.68–6.32 h/day) (p < 0.01). Worldwide, the group with a mean scan rate of 3.4 spent 10.77 h/day in hyperglycemia (95% CI 10.71–10.83 h/day), while the group with a mean scan rate of 37.8 spent 5.82 h in hyperglycemia (95% CI 5.77–5.87 h/day) (p < 0.01). The time spent in hyperglycemia of the bin with the lowest scan rate was clinically and significantly lower in Brazil compared to the rest of the world (2.11 h/day lower, p < 0.01). However, there were no significant differences between the time spent in hyperglycemia of the bin with the highest scan rate for Brazil compared to the rest of the world (p = 0.29).

### Time in range

**Fig. 4:**
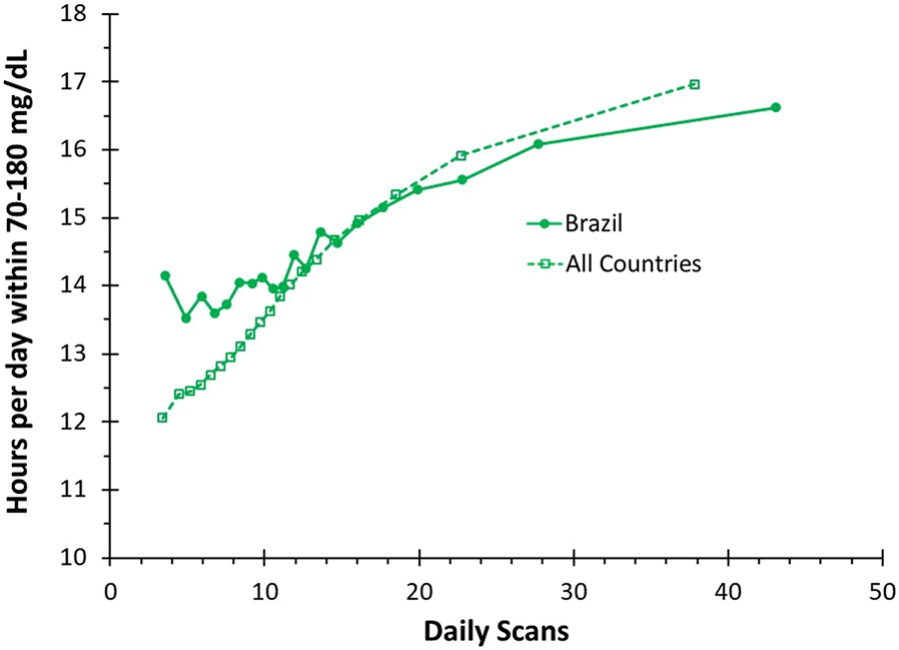
Time within 70–180 mg/dL: a comparison of mean time in range per day by number of daily scans for CGM users worldwide compared to users in Brazil

In both Brazil and around the world, as scan frequency increased time in range increased (Fig. 4). In Brazil, from the lowest to the highest scan frequency group, time in range increased from 14.15 to 16.62 h/day (p < 0.01). Worldwide, from the lowest to the highest scan group time in range increased from 12.06 to 16.97 h/day (p < 0.01). The time in range for the bin with the lowest scan rate was clinically and significantly higher in Brazil compared to the rest of the world (p < 0.01).

### Time in hypoglycemia

**Fig. 5:**
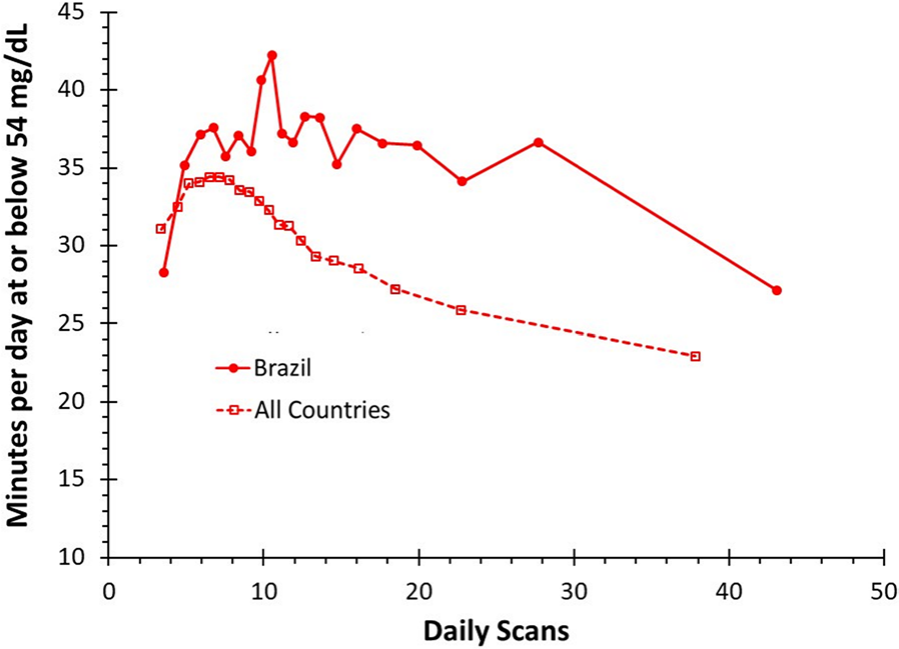
Time at or below 54 mg/dL: a comparison of mean minutes per day in hypoglycemia by number of daily scans for CGM users worldwide compared to users in Brazil

In Brazil, the group with the lowest scan frequency spent an average of 28.25 min per day (95% CI 25.02–31.48 min per day) in hypoglycemia (≤ 54 mg/dL) while the group with the highest scan frequency spent 27.14 (95% CI 23.83–30.46 min per day) minutes per day in hypoglycemia (p = 0.64). Worldwide, the group with the lowest scan frequency spent an average of 31.08 min per day (95% CI 30.56–31.60 min per day) in hypoglycemia while the group with the highest scan frequency spent 22.93 min per day (95% CI 22.46–23.40 min per day) in hypoglycemia (p < 0.01) (Fig. 5).

## Discussion

This was the first study to investigate the comparative association of sensor-based glucose monitoring on glycemic markers in developing countries like Brazil. Like users worldwide [20], users in Brazil also demonstrated that increased scan frequency is associated with lower estimated A1c, decreased time in hyperglycemia, and increased time in range. Brazil users had a higher mean scan rate compared to users around the world. The increased scan frequency was associated with improved glycemic outcomes compared to the rest of the world in the bins with the lowest scan frequency. No significant differences were observed between Brazil users and world users for the bin with the highest scan frequency.

It is interesting to note that time in hypoglycemia increased from lower scan groups until around 7 scans/day, before starting to decrease. Around the world, as scan frequency increased the number of minutes spent in hypoglycemia increased to 34.42 min per day (95% CI 33.94–34.91 min per day) for a scan frequency 7.15 scans per day (bin 7) before decreasing. Previous studies have demonstrated that increased occurrence of hypoglycemia is associated with increased glycemic variability but not HbA1c or mean glucose [21, 22]. In the current study, the coefficient of variation increased from 38% for the lowest scan rate group to 40% for 7.15 scans per day (bin 7) before decreasing to 34% for the highest scan group. Thus, the observed increase in hypoglycemia at approximately 7 scans per day is associated with a decrease in time spent in hyperglycemia as indicated by Fig. 3 but with an associated increase in glycemic variability, thus leading to increased hypoglycemia in these groups. Above 7 scans/day there is a progressive trend to reduction of hypoglycemia, suggesting that maybe there is a boundary of number of scans required to transfer information to glucose metrics. It is also important to address that in the TIR consensus it is stressed that more than 70% of CGM use over the most recent 14 days correlates strongly with 3 months of mean glucose, but for T1D patients the correlations are weaker for hypoglycemia and glycemic variability, thus confirming that these two metrics are more challenging to interpret [8].

This study has two key strengths. (1) A large number of readers are analyzed with consistent technology and methodology making it possible to compare individual country numbers with world data. (2) The results capture real-world data for CGM users obtained without any experimental intervention. Over 688,000 readers from around the world and over 17,000 readers from Brazil are included in this analysis. Despite the large volume of data, little information is available regarding specific characteristics of the users, including type of diabetes, pump usage, and age. Moreover, because continuous glucose monitoring is reimbursed in some countries, but not in others, the constitution and motivations of the user population may differ from one country to another. Nonetheless, the data from both the developing country of Brazil and data from around the world unequivocally demonstrate that increased scan frequency is associated with improved glycemic control. This observation is important because it suggests that a database like this can eventually reduce the risk of bias related to specific parameters of the studied populations. More studies like this, with different populations, would help us confirm this impression.

### Significance of these results in relation to Brazilian culture

The scan frequency of Brazilian users was greater than that of users worldwide. One potential explanation for this difference is that Brazil has the third largest population of children and adolescents with type 1 diabetes in the world, behind the USA and India [14]. Another factor may be that Brazilian users paid for CGMs “out of pocket” whereas CGMs are more universally accessible in other countries. Thus, the Brazilian dataset may be selected towards children and adolescents, individuals with increased diabetes self-management education, and means to purchase CGMs. This aligns with the observation that Brazil has improved glycemic outcomes for comparable scan frequencies in the lower scan rate groups compared to the rest of the world.

## Conclusions

The present study suggests that Brazilian flash glucose monitor users have a high frequency of glucose scans and that increased scan frequency is associated with better glycemic control. Compared to the findings observed in global population, Brazilians scan more frequently than their counterparts worldwide, but the data from both databases is similar, and unequivocally demonstrate that increased scan frequency is associated with improved glycemic control. Quantitative studies are needed to better understand the importance of population characteristics on the scan frequency worldwide, and also among the Brazilian population and how this subpopulation compares to the Brazilian population at large.

## Data Availability

The data that support the findings of this study are available from Abbott Diabetes Care but restrictions apply to the availability of these data and are not publicly available. Data are however available from the authors upon reasonable request and with permission of Abbott Diabetes Care.

## References

[1] American Diabetes Association (2019). 6. Glycemic targets: standards of medical care in diabetes—2019. Diabetes Care..

[2] Alexander CM, Amiel S, Beck R, Bergenstal RM, Bloomgarden Z, Brown A (2018). Need for regulatory change to incorporate beyond A1C glycemic metrics. Diabetes Care.

[3] Bergenstal RM, Gal RL, Connor CG, Gubitosi-Klug R, Kruger D, Olson BA (2017). Racial differences in the relationship of glucose concentrations and hemoglobin A1c levels. Ann Intern Med.

[4] Danne T, Nimri R, Battelino T, Bergenstal RM, Close KL, DeVries JH (2017). International consensus on use of continuous glucose monitoring. Diabetes Care.

[5] Beck RW, Bergenstal RM, Riddlesworth TD, Kollman C, Li Z, Brown AS, Close KL (2019). Validation of time in range as an outcome measure for diabetes clinical trials. Diabetes Care.

[6] Lu J, Ma X, Zhou J, Zhang L, Mo Y, Ying L (2018). Association of time in range, as assessed by continuous glucose monitoring, with diabetic retinopathy in type 2 diabetes. Diabetes Care.

[7] American Diabetes Association (2019). 7. Diabetes technology: standards of medical care in diabetes—2019. Diabetes Care.

[8] Battelino T, Danne T, Bergenstal RM, Amiel SA, Beck R, Biester T (2019). Clinical targets for continuous glucose monitoring data interpretation: recommendations from the international consensus on time in range. Diabetes Care.

[9] Miller KM (2013). Evidence of a strong association between frequency of self-monitoring of blood glucose and hemoglobin A1c levels in T1D exchange clinic registry participants. Diabetes Care.

[10] Dunn TC, Xu Y, Hayter G, Ajjan RA (2018). Real-world flash glucose monitoring patterns and associations between self-monitoring frequency and glycaemic measures: a European analysis of over 60 million glucose tests. Diabetes Res Clin Pract.

[11] Heinemann L (2008). Finger pricking and pain: a never ending story. J Diabetes Sci Technol.

[12] Bolinder J, Antuna R, Geelhoed-Duijvestijn P, Kröger J, Weitgasser R (2016). Novel glucose-sensing technology and hypoglycaemia in type 1 diabetes: a multicentre, non-masked, randomised controlled trial. Lancet.

[13] Jangam S, Dunn T, Xu Y, Hayter G, Ajjan RA (2019). Flash glucose monitoring improves glycemia in higher risk patients: a longitudinal, observational study under real-life settings. BMJ Open Diabetes Res Care.

[14] Karuranga S, da Rocha Fernandes HY, Malanda B (2018). IDF diabetes atlas.

[15] Aivalikli J. From slums to sustainability? Internal migration in a highly urbanised developing country: the case of Brazil. 2018.

[16] Wagner FE, Ward JO (1980). Urbanization and migration in Brazil. Am J Econ Sociol.

[17] King H, Aubert RE, Herman WH (1998). Global burden of diabetes, 1995–2025: prevalence, numerical estimates, and projections. Diabetes Care.

[18] International Hypoglycaemia Study Group (2017). Glucose concentrations of less than 3.0 mmol/L (54 mg/dL) should be reported in clinical trials: a joint position statement of the American Diabetes Association and the European Association for the Study of Diabetes. Diabetes Care.

[19] Nathan DM, Kuenen J, Borg R, Zheng H, Schoenfeld D, Heine RJ (2008). Translating the A1C assay into estimated average glucose values. Diabetes Care.

[20] Lang J, Jangam S, Dunn T, Hayter G. Expanded real-world use reaffirms strong correlation between scanning frequency of flash glucose monitoring and glucose control. 2019. 972p.

[21] Weinstock RS, DuBose SN, Bergenstal RM, Chaytor NS, Peterson C, Olson BA, Munshi MN, Perrin AJ, Miller KM, Beck RW, Liljenquist DR (2016). Risk factors associated with severe hypoglycemia in older adults with type 1 diabetes. Diabetes Care.

[22] Oskarsson P, Antuna R, Geelhoed-Duijvestijn P, Krӧger J, Weitgasser R, Bolinder J (2018). Impact of flash glucose monitoring on hypoglycaemia in adults with type 1 diabetes managed with multiple daily injection therapy: a pre-specified subgroup analysis of the IMPACT randomised controlled trial. Diabetologia.

